# Enhanced levels of leukotriene B_4_ in synovial fluid in Lyme disease

**DOI:** 10.1155/S0962935193000304

**Published:** 1993

**Authors:** E. Mayatepek, D. Hassler, M. Maiwald

**Affiliations:** 1 University Children's Hospital Im Neuenheimer Feld 150 Heidelberg 6900 Germany; 2 Untere Hofstatt Kraichtal 7527 Germany; 3 Institute of Hygiene Im Neuenheimer Feld 324 Heidelberg 6900 Germany

## Abstract

The purpose of this study was to evaluate the potential role of LTB_4_ and cysteinyl leukotrienes in Lyme disease (LD). Therefore, a total number of 34 patients divided into four groups was studied. The patients were classified as having Lyme arthritis (n = 7) or Lyme meningitis (n = 10), and as control groups patients with a noninflammatory arthropathy (NIA) (n = 7) and healthy subjects (n = 10). LTB_4_ as well as LTC_4_ secretion from stimulated polymorphonuclear leukocytes (PMNL) from all groups of patients showed no statistical differences. LTB_4_ levels in synovial fluid were significantly increased in patients with Lyme arthritis (median 142 ng/ml, range 88–296) when compared to the control subjects with NIA (median 46 ng/ml, range 28–72) (p < 0.05). No statistical difference of urinary LTE_4_ levels between all the different groups of patients was observed. These results show that cysteinyl leukotrienes do not play an important role in the pathogenesis of LD. In contrast to previous findings in rheumatoid arthritis, LTB_4_ production from stimulated PMNL was not found to be increased in LD. However, the significantly elevated levels of LTB_4_ in synovial fluid of patients with Lyme arthritis underline the involvement of LTB_4_ in the pathogenesis of this disease.


Mediators of Inflammation, 2, 225–228 (1993)

### Enhanced levels of leukotriene $B_4$ in synovial fluid in Lyme disease

E. Mayatepek,[1,CA] D. Hassler,[2] and M. Maiwald[3]

[1]University Children's Hospital, Im Neuenheimer Feld 150, 6900 Heidelberg, Germany; [2]Untere Hofstatt, 7527 Kraichtal, Germany; [3]Institute of Hygiene, Im Neuenheimer Feld 324, 6900 Heidelberg, Germany

[CA] Corresponding Author

**THE purpose of this study was to evaluate the potential role of $LTB_4$ and cysteinyl leukotrienes in Lyme disease (LD). Therefore, a total number of 34 patients divided into four groups was studied. The patients were classified as having Lyme arthritis ($n = 7$) or Lyme meningitis ($n = 10$), and as control groups patients with a non-inflammatory arthropathy (NIA) ($n = 7$) and healthy subjects ($n = 10$). $LTB_4$ as well as $LTC_4$ secretion from stimulated polymorphonuclear leukocytes (PMNL) from all groups of patients showed no statistical differences. $LTB_4$ levels in synovial fluid were significantly increased in patients with Lyme arthritis (median 142 ng/ml, range 88–296) when compared to the control subjects with NIA (median 46 ng/ml, range 28–72) ($p < 0.05$). No statistical difference of urinary $LTE_4$ levels between all the different groups of patients was observed. These results show that cysteinyl leukotrienes do not play an important role in the pathogenesis of LD. In contrast to previous findings in rheumatoid arthritis, $LTB_4$ production from stimulated PMNL was not found to be increased in LD. However, the significantly elevated levels of $LTB_4$ in synovial fluid of patients with Lyme arthritis underline the involvement of $LTB_4$ in the pathogenesis of this disease.**

**Key words:** Arthritis, Cysteinyl leukotrienes, $LTB_4$, Lyme disease

### Introduction

Lyme disease (LD) is a multisystem infection caused by the spirochete *Borrelia burgdorferi* which is transmitted by *Ixodes* ticks.[1–3] The disease is associated with a variety of clinical manifestations which include erythema migrans, lymphocytic meningitis, motor or sensory radiculitis and, particularly in later stages, involvement of the joints in the form of asymmetrical mono- or oligoarthritis.[4,5]

Cytokines, such as interleukin-1, interleukin-6 or tumour necrosis factor, have been implicated in the pathophysiology of LD.[6,7] However, the potential role of other mediators of inflammation such as leukotrienes in LD has not received a great deal of attention. Leukotriene $B_4$ ($LTB_4$), one of the most powerful chemotactic and chemokinetic agents,[8] has been found to exert strong leukocytotropic activities and can cause neutrophil degranulation.[9] It is readily synthesized by phagocytic cells, principally neutrophils[10] and macrophages[11] on challenge with a variety of stimuli. The cysteinyl leukotrienes ($LTC_4$, $LTD_4$, $LTE_4$), however, increase microvascular permeability, induce symptoms of smooth muscle contraction and cause oedema.[12–15]

Increased $LTB_4$ production by stimulated polymorphonuclear leukocytes (PMNL) from patients with rheumatoid arthritis as well as elevated $LTB_4$ in synovial tissues in rheumatoid arthritis and spondyloarthritis has already been demonstrated.[16–18] Elevated urinary $LTE_4$ levels have been reported in patients with active systemic lupus erythematosus, a connective tissue disease, characterized by marked immunological abnormalities leading to inflammation and tissue injury.[19] These data also suggest an involvement of leukotrienes in human inflammatory disease such as LD.

The aim of this study was therefore to evaluate the potential role of $LTB_4$ and cysteinyl leukotrienes in patients with Lyme arthritis and Lyme meningitis in comparison to patients with noninflammatory arthropathy and healthy subjects. $LTB_4$ as well as $LTC_4$ production was quantified in stimulated PMNL; moreover, $LTB_4$ levels were determined in synovial fluid and additionally $LTE_4$ was measured in the urine of all patients.

### Materials and Methods

*Patients:* A total of 34 patients was entered into this study after informed consent was obtained.

© 1993 Rapid Communications of Oxford Ltd

The patients were classified as having either (i) meningitis due to an infection with *B. burgdorferi* with a characteristic immunoblot pattern and intrathecally produced antibodies ($n = 10$); (ii) mono- or oligoarthritis serologically positive to *B. burgdorferi* by characteristic immunoblot pattern ($n = 7$); or (iii) patients with a noninflammatory arthropathy (NIA) such as degenerative or traumatic joint disease ($n = 7$) as a control group to Lyme arthritis; and (iv) a healthy control group ($n = 10$). None of the patients received any medication before the study and all had a normal liver and renal function. In all patients other bacterial, viral or rheumatological diseases could be excluded.

*Serological assays for diagnosis of Lyme disease:* The indirect immunofluorescence assay, quantitative enzyme immunoassay (EIA) and immunoblot were performed as described previously.[20,21] Cut-off titres were 256 (IgG) or 32 (IgM), respectively, for IFA and 200 standard units (IgG) for EIA. CSF antibody concentrations were measured by means of the quantitative EIA using a cut-off value of 2.0 standard units. Diagnosis of LD was established in each patient by characteristic immunoblot according to the criteria described previously.[20,22]

*Quantification of $LTB_4$ and $LTC_4$ in stimulated PMNL:* Heparinized venous blood was obtained from all patients studied. After removal of mononuclear cells by Ficoll–Hypaque density gradient centrifugation, the neutrophil-rich pellet was sedimented by dextran. Residual erythrocytes were lysed by hypotonic saline (0.45%). Purity and viability of the neutrophil suspension as assessed by Trypan blue exclusion was consistently more than 95%. Activation of isolated PMNL with calcium ionophore A23187 (10 $\mu$M; Sigma Chemical Co., St Louis, MO) was carried out as described.[23] $^3$H-labelled $LTB_4$ or $^3$H-labelled $LTC_4$ (both Du Pont–New England Nuclear, Boston, MA) was added as an internal standard. Samples were pumped through activated Sep-Pak C18 cartridges (Waters, Milford, MA). The cartridges were washed with distilled water and eluted with 5 ml 90% aqueous methanol containing 1 mM 4-hydroxy-2,2,6,6-tetramethylpiperidine-N(1)-oxyl (HTMP; Sigma Chemicals Co., St Louis, MO) and 0.5 mM EDTA. The eluates were evaporated to dryness under reduced pressure and resuspended in 30% ice-cold aqueous methanol. The samples were then injected into a C18 reversed-phase high-performance liquid chromatography (RP-HPLC) column (Shandon, Runcorn, UK) and eluted through a HPLC system (Knauer, Berlin, FRG) at a constant flow rate of 1 ml/min, using a mixture of acetonitrile/water (38:62 vol/vol) the aqueous part containing 0.1% acetic acid, 1 mM EDTA, and adjusted to pH 5.6 by ammonium hydroxide for separation of $LTB_4$. $LTC_4$ fractions were prepared isocratically with a methanol/water (65:35 vol/vol) system, the aqueous part showing the identical composition as described above. The fractions having the same elution time as the synthetic $LTB_4$ or $LTC_4$ were collected and immunoreactive $LTB_4$ as well as $LTC_4$ content was determined by EIA (Cayman, Ann Arbor, MA).

*Measurement of $LTB_4$ in synovial fluid:* Synovial fluid was aspirated in all patients with Lyme arthritis and noninflammatory arthropathy as part of diagnostic procedures from knee joints and centrifuged at $2\,000 \times g$ for 10 min to remove cells and particulate material; the supernatant fluid was stored at $-80°C$ until $LTB_4$ was extracted. After $^3$H-labelled $LTB_4$ was added to each 2–3 ml sample of synovial fluid, the samples were titrated to pH 4.0 with 2 M citric acid. Each sample was extracted three times with 4 ml of chloroform:methanol (2:1 vol/vol), the organic phases were pooled and dried under nitrogen. $LTB_4$ was resolved and purified by Sep-Pak C18 extraction and RP-HPLC as described above. Quantification of $LTB_4$ was performed by EIA as stated above.

*Urine $LTE_4$ analysis:* Urine was obtained from spontaneous micturition and mixed with two volumes of 90% (vol/vol) aqueous methanol of pH 8.5 containing 0.5 mM EDTA, 1 mM HTMP, and 20 mM $KHCO_3$, and stored at $-80°C$ under argon until later use. Aliquots of each urine sample were screened to exclude the presence of pathological constituents. Urinary $LTE_4$ was measured essentially as described elsewhere.[24] Urine samples were tested in duplicate. Samples were allowed to thaw immediately before the assay. $^3$H-labelled $LTE_4$ (Du Pont–New England Nuclear, Boston, MA) was added as an internal standard. Samples were then acidified to pH 4.5 by addition of 0.1 M HCl, homogenized, and pumped through activated Sep-Pak cartridges as already described. Fractions having the same elution time as the synthetic $LTE_4$ were separated by RP-HPLC as described above for $LTC_4$. The immunoreactive $LTE_4$ content was determined by EIA (Cayman, Ann Arbor, MA).

*Statistical analysis:* The Wilcoxon–Mann–Whitney test for the one-sided problem was used for statistical comparison between the different groups of patients. Differences were considered significant when $p$ was less than 0.05.

### Results

*$LTB_4$ and $LTC_4$ secretion from stimulated PMNL:* $LTB_4$ as well as $LTC_4$ secretion from stimulated PMNL from all groups of patients is shown in

**Table 1.** $LTB_4$ and $LTC_4$ production from stimulated polymorphonuclear leukocytes from patients with Lyme arthritis, noninflammatory arthropathy (NIA), Lyme meningitis, and healthy subjects. Values are given as the median with the range in brackets

| Leukotriene (ng per $10^6$ cells) | Lyme arthritis ($n = 7$) | NIA ($n = 7$) | Lyme meningitis ($n = 10$) | Healthy subjects ($n = 10$) |
|---|---|---|---|---|
| $LTB_4$ | 40.0 (34.2–47.1) | 38.4 (33.4–44.8) | 39.3 (32.0–45.9) | 35.2 (33.4–44.6) |
| $LTC_4$ | 4.8 (4.1–5.6) | 4.6 (4.0–5.3) | 5.3 (4.4–5.8) | 5.0 (3.9–5.5) |

Table 1. No statistical difference of $LTB_4$ as well as $LTC_4$ production between all groups of patients was observed.

*$LTB_4$ in synovial fluid:* $LTB_4$ was detected in all synovial fluid samples tested. $LTB_4$ levels in synovial fluid were significantly increased in patients with Lyme arthritis (median 142 ng/ml, range 88–296) when compared to the control subjects with NIA (median 46 ng/ml, range 28–72) ($p < 0.05$) (Fig. 1). Although the total white blood cell counts in synovial fluid were higher in the patients with Lyme arthritis than in NIA, no significant correlation was found between the white blood cell count and the $LTB_4$ levels in synovial fluid of patients with Lyme arthritis ($r = 0.14$) or with NIA ($r = 0.15$).

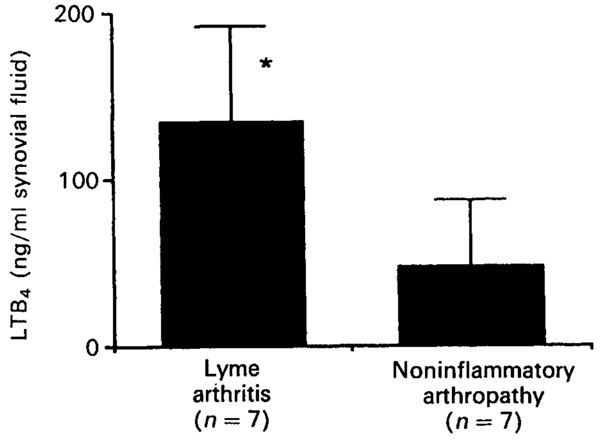

FIG. 1. $LTB_4$ levels in synovial fluid of patients with Lyme arthritis or noninflammatory arthropathy (NIA). Values are expressed as mean ± S.E.M. *$p < 0.05$ as compared to patients with NIA.

*$LTE_4$ in urine:* Urinary $LTE_4$ levels of all patients are given in pmol/l as well as in nmol/mol creatinine (Table 2). No statistical difference of $LTE_4$ levels between the different groups of patients was observed.

### Discussion

The present data indicate that the synovial levels of the lipoxygenase product $LTB_4$ is elevated in Lyme arthritis compared with the levels in synovia of subjects with NIA (Fig. 1). Similar results of increased synovial $LTB_4$ levels have been reported only in rheumatoid arthritis.[17,18] $LTB_4$ is chemotactic for neutrophils and eosinophils *in vitro* at a concentration as low as 3 ng/ml and evokes a maximal chemotactic response at 30 ng/ml.[25] Thus, the concentration of $LTB_4$ in synovial fluid may be sufficient to contribute to the local inflammatory reaction. Because the involvement of $LTB_4$ in several T-cell activation stages, namely proliferation, induction of helper and suppressor function and production of interleukin-2, interferon-$\gamma$[26] and interleukin-1,[27] $LTB_4$ might contribute to the pathogenesis of Lyme arthritis.

In contrast to synovial fluid and previous findings of elevated $LTB_4$ production from PMNL in rheumatoid arthritis,[16] the $LTB_4$ secretion from stimulated PMNL was detected at similar levels in patients with Lyme arthritis, NIA, Lyme meningitis, and healthy controls (Table 1). This demonstrates that peripheral blood leukocytes do not produce increased quantities of $LTB_4$ in LD. These results suggest that local but not systemic

**Table 2.** $LTE_4$ in urine of patients with arthritis or meningitis due to an infection with *B. burgdorferi* (Lyme disease), noninflammatory arthropathy (NIA) and healthy subjects. Values are given as the median with the range in brackets

| $LTE_4$ (concentration) | Lyme arthritis ($n = 7$) | NIA ($n = 7$) | Lyme meningitis ($n = 10$) | Healthy subjects ($n = 10$) |
|---|---|---|---|---|
| pmol/l | 279 (89–476) | 262 (72–435) | 273 (95–465) | 265 (70–430) |
| nmol/mol creatinine | 25 (18–62) | 20 (15–54) | 23 (16–56) | 19 (11–48) |

production of $LTB_4$ could account for symptoms of arthritis in late LD.

Although isolation of *B. burgdorferi* is very difficult in Lyme arthritis it has been achieved in some cases from synovial fluid years after onset.[28] Up to now, it is not clear whether autoreactivity as well as increased synovial $LTB_4$ is an epiphenomenon of persistent infection or is an important factor in tissue damage. The mechanisms underlying this process are a prospect for future research. An exact knowledge of the role of $LTB_4$ in the pathogenetic contribution in Lyme arthritis may yield new therapeutic approaches, e.g. in the (local) application of selective inhibitors or antagonists.

The results also show that cysteinyl leukotrienes are not enhanced when generated in LD. $LTC_4$ production from PMNL in patients with LD did not differ from those measured in NIA and healthy subjects (Table 1). Urinary $LTE_4$ has been proposed and used as the index metabolite for the systemic generation of cysteinyl leukotrienes in humans.[19,29–31] Urine $LTE_4$ levels in patients with Lyme arthritis or Lyme meningitis, however, were not significantly different from patients with NIA or healthy controls (Table 2). Recent data indicate that increased synthesis of leukotrienes as measured by a rise in urinary $LTE_4$ levels is associated with active systemic lupus erythematosus and scleroderma and suggest that cysteinyl leukotrienes may mediate certain symptoms associated with these diseases.[19] It seems possible that elevated $LTE_4$ levels were not seen in our patients with Lyme arthritis due to the more localized nature of the inflammatory process. As in rheumatoid arthritis where $LTE_4$ levels were also found to be normal,[19] most inflammation is localized to the synovium.

In summary, the results show that cysteinyl leukotrienes do not play an important role in the pathogenesis of LD. $LTB_4$ production from stimulated PMNL was not found to be increased in LD. However, the significantly elevated levels of $LTB_4$ in synovial fluid in patients with Lyme arthritis underline the involvement of $LTB_4$ in the pathogenesis of this disease.

### References

1. Burgdorfer W, Barbour AC, Hayes SF, *et al.* Lyme disease: a tick-borne spirochetosis? *Science* 1982; **216:** 1317–1319.
2. Benach JL, Bosler EM, Hanrahan JP, *et al.* Spirochetes isolated from the blood of two patients with Lyme disease. *N Engl J Med* 1983; **308:** 740–742.
3. Steere AC, Grodzicki RL, Kornblatt AN, *et al.* The spirochetal etiology of Lyme disease. *N Engl J Med* 1983; **308:** 733–740.
4. Steere AC, Malawista SE, Syndman DR, *et al.* Lyme arthritis: an epidemic of oligoarticular arthritis in children and adults in three Connecticut communities. *Arthritis Rheum* 1977; **20:** 7–17.
5. Steere AC. Lyme disease. *N Engl J Med* 1989; **321:** 586–596.
6. Habicht GS, Beck G, Benach JL, Coleman JL, Leichtling KD. Lyme disease spirochetes induce human and murine interleukin-1 production. *J Immunol* 1985; **134:** 147–154.
7. Habicht GS, Katona LI, Benach JL. Cytokines and the pathogenesis of neuroborreliosis: *Borrelia burgdorferi* induces glioma cells to secrete interleukin-6. *J Infect Dis* 1991; **164:** 568–574.
8. Ford-Hutchinson AW, Bray MA, Doig MV, Shipley ME, Smith MJ. Leukotriene B, a potent chemokinetic and aggregating substance released from polymorphonuclear leukocytes. *Nature* 1980; **286:** 264–265.
9. Showell HJ, Naccache PH, Borgeat P, *et al.* Characterization of the secretory activity of $LTB_4$ toward rabbit neutrophils. *J Immunol* 1982; **128:** 811–816.
10. Borgeat P, Samuelsson B. Metabolism of arachidonic acid in polymorphonuclear leukocytes: effect of the ionophore A23187. *Proc Natl Acad Sci USA* 1979; **76:** 2148–2152.
11. Fels AO, Pawloski NA, Cramer EB, King TK, Cohen ZA, Scott WA. Human alveolar macrophages produce leukotriene $B_4$. *Proc Natl Acad Sci USA* 1982; **79:** 7866–7870.
12. Murphy RC, Hammarström S, Samuelsson B. Leukotriene C: a slow-reacting substance from murine mastocytoma cells. *Proc Natl Acad Sci USA* 1979; **76:** 4275–4279.
13. Hammarström S. Leukotrienes. *Annu Rev Biochem* 1983; **52:** 355–377.
14. Lewis RA, Austen KF. The biologically active leukotrienes. *J Clin Invest* 1984; **73:** 889–897.
15. Samuelsson B, Dahlén S-E, Lindgren JA, Rouzer CA, Serhan CN. Leukotrienes and lipoxins: structures, biosynthesis, and biological effects. *Science* 1987; **237:** 1171–1176.
16. Belch JJ, O'Dowd A, Ansell D, Sturrock RD. Leukotriene $B_4$ production by peripheral blood neutrophils in rheumatoid arthritis. *Scand J Rheumatol* 1989; **18:** 13–19.
17. Klickstein LB, Shapleigh C, Goetzl EJ. Lipoxygenation of arachidonic acid as a source of polymorphonuclear leukocyte chemotactic factors in synovial fluid and tissue in rheumatoid arthritis and spondyloarthritis. *J Clin Invest* 1980; **66:** 1166–1170.
18. Davidson EM, Rae SA, Smith MJ. Leukotriene $B_4$, a mediator of inflammation present in synovial fluid in rheumatoid arthritis. *Ann Rheum Dis* 1983; **43:** 677–679.
19. Hackshaw KV, Voelkel NF, Thomas RB, Westcott JY. Urine leukotriene $E_4$ levels are elevated in patients with active systemic lupus erythematosus. *J Rheumatol* 1992; **19:** 252–258.
20. Zöller L, Burkard S, Schäfer H. Validity of Western immunoblot band patterns in the serodiagnosis of Lyme borreliosis. *J Clin Microbiol* 1991; **29:** 174–182.
21. Zöller L, Haude M, Hassler D, Burkard S, Sonntag HG. Spontaneous and post-treatment antibody kinetics in late Lyme borreliosis. *Serodiagn Immunoth Infect Dis* 1989; **3:** 345–353.
22. Mayatepek E, Neuß M, Zöller L. Serological and clinical findings in childhood Lyme borreliosis. *Immunol Infect Dis* 1991; **1:** 335–339.
23. Weller PF, Lee CW, Foster DW, Corey EJ, Austen KF, Lewis RA. Generation and metabolism of 5-lipoxygenase pathway leukotrienes by human eosinophils: predominant production of leukotriene $C_4$. *Proc Natl Acad Sci USA* 1983; **80:** 7626–7630.
24. Huber M, Müller J, Leier I, *et al.* Metabolism of cysteinyl leukotrienes in monkey and man. *Eur J Biochem* 1990; **194:** 309–315.
25. Goetzl EJ, Pickett WC. The human PMN leukocyte chemotactic activity of complex hydroxy-eicosatetraenoic acids (HETEs). *J Immunol* 1980; **125:** 1789–1791.
26. Rola-Pleszczynski M, Chavaillaz PA, Lemaire I. Stimulation of interleukin 2 and interferon-γ production by leukotriene $B_4$ in human lymphocyte cultures. *Prostaglandins Leukot Med* 1986; **23:** 207–210.
27. Rola-Pleszczynski M, Lemaire I. Leukotrienes augment interleukin 1 production by human monocytes. *J Immunol* 1985; **135:** 3958–3961.
28. Huppertz H-I. Childhood Lyme borreliosis in Europe. *Eur J Pediatr* 1990; **149:** 814–821.
29. Keppler D, Huber M, Hagmann W, Ball HA, Guhlmann A, Kästner S. Metabolism and analysis of endogenous cysteinyl leukotrienes. *Ann NY Acad Sci* 1988; **524:** 68–74.
30. Tayor GW, Taylor I, Black P, *et al.* Urinary leukotriene $E_4$ after antigen challenge in acute asthma and allergic rhinitis. *Lancet* 1989; **i:** 584–588.
31. Huber M, Kästner S, Schölmerich J, Gerok W, Keppler D. Analysis of cysteinyl leukotrienes in human urine: enhanced excretion in patients with liver cirrhosis and hepatorenal syndrome. *Eur J Clin Invest* 1989; **19:** 53–60.

ACKNOWLEDGEMENTS. We thank Dr G. Pecher, Rostock, Germany, for kindly providing some of the patients' samples. This paper is dedicated to Professor H. J. Bremer on the occasion of his 60th birthday

**Received 20 January 1993; accepted in revised form 22 March 1993.**
